# Observed rates of surgical instrument errors point to visualization tasks as being a critically vulnerable point in sterile processing and a significant cause of lost chargeable OR minutes

**DOI:** 10.1186/s12893-024-02407-1

**Published:** 2024-04-15

**Authors:** Peter F. Nichol, Mark J. Saari, Natalia Navas, David Aguilar, Rita K. Bliesner, Paige J. Brunner, Jacob C. Caceres, Madelyn Chen, Ava R. VanDommelen, Matthew Fischer, Simar Garcha, Elaf A. Ghawas, Grace R. Hackinson, Ava Hitzeman, Maria Jabbour, Amanda M. Jentsch, Madison M. Kurth, Mollyn Leyden, Qianyun Luo, Abigail C. McGrain, Gwendolyn Nytes, Olivia R. O’Brien, Jesibell K. Philavong, Natalie Villegas, Shannon R. Walsh, Sydney S. Wisdorf

**Affiliations:** 1 Department of Surgery, University of Wisconsin School of Medicine, and Public Health, H4/746 CSC 600 Highland Ave. Madison, Madison, WI 53792 USA; 2 University of Wisconsin School of Medicine, and Public Health, Madison, USA; 3 Arizona State University, Tempe, USA; 4 University of Wisconsin-Madison, Madison, USA

**Keywords:** Sterile processing, Surgical instrument errors, Cost of an OR minute, Human error, Visualization technologies, Chargeable OR minutes

## Abstract

**Background:**

The reporting of surgical instrument errors historically relies on cumbersome, non-automated, human-dependent, data entry into a computer database that is not integrated into the electronic medical record. The limitations of these reporting systems make it difficult to accurately estimate the negative impact of surgical instrument errors on operating room efficiencies. We set out to determine the impact of surgical instrument errors on a two-hospital healthcare campus using independent observers trained in the identification of Surgical Instrument Errors.

**Methods:**

This study was conducted in the 7 pediatric ORs at an academic healthcare campus. Direct observations were conducted over the summer of 2021 in the 7 pediatric ORs by 24 trained student observers during elective OR days. Surgical service line, error type, case type (inpatient or outpatient), and associated length of delay were recorded.

**Results:**

There were 236 observed errors affecting 147 individual surgical cases. The three most common errors were Missing+ (*n* = 160), Broken/poorly functioning instruments (*n* = 44), and Tray+ (*n* = 13). Errors arising from failures in visualization (i.e. inspection, identification, function) accounted for 88.6% of all errors (Missing+/Broken/Bioburden). Significantly more inpatient cases (42.73%) had errors than outpatient cases (22.32%) (*p* = 0.0129). For cases in which data was collected on whether an error caused a delay (103), over 50% of both IP and OP cases experienced a delay. The average length of delays per case was 10.16 min. The annual lost charges in dollars for surgical instrument associated delays in chargeable minutes was estimated to be between $6,751,058.06 and $9,421,590.11.

**Conclusions:**

These data indicate that elimination of surgical instrument errors should be a major target of waste reduction. Most observed errors (88.6%) have to do with failures in the visualization required to identify, determine functionality, detect the presence of bioburden, and assemble instruments into the correct trays. To reduce these errors and associated waste, technological advances in instrument identification, inspection, and assembly will need to be made and applied to the process of sterile processing.

**Supplementary Information:**

The online version contains supplementary material available at 10.1186/s12893-024-02407-1.

## Background

The study of surgical instrument errors as well as the process of sterile processing are relatively nascent fields. As of December 2, 2023, there were only 194 peer-reviewed publications when entering the search term “sterile processing” in PubMed. Recent studies indicate that staff reporting of these problems is burdensome, frequently incomplete, delayed, and underreports the extent of the problem [1]. Because of the low rate by which surgical instrument errors are reported by staff, it is difficult to accurately estimate the real-world rate of surgical instrument errors and its financial impact on healthcare systems. Without being able to accurately estimate the extent of this problem and the financial impact on efficiencies, there is little to persuade healthcare leaders that the current state of sterile processing is resulting in a sub-optimal outcome in the delivery of surgical care.

That limits in human performance can adversely affect the preparation of surgical instruments during sterile processing should not be surprising. The study of the limits human performance has been an area of deep inquiry in the high-end manufacturing space for decades. These limits are nicely described in David Smith’s textbook, Maintainability, Reliability and Risk 6th Ed [2]. Essentially, risk of error increases with complexity of task and stress in the work environment. Until recently, sterile processing has not been examined through this lens. Error modeling of the Surgical Instrument Cycle, which is the series of tasks required to render a surgical instrument sterile and in proper working order for the next case after use in the OR, points to complicated, non-routine tasks involving visualization (inspection for bioburden, function, and identification for sorting) as being at greatest risk for error [3]. Staff reporting of surgical instrument errors supports this modeling as the majority of reported errors (83%) arise from failed inspection (bioburden and function) and identification/sorting (missing instruments) [1].

We undertook a direct observation study to measure the rate of surgical instrument errors. Our goals were three-fold: first, To test the hypothesis that visualization tasks in sterile processing are the most vulnerable to errors. If the data supports our hypothesis, then the majority of errors would fall in to the Missing, Broken/poorly functioning instrument, and Bioburden categories. Our second goal was to establish an institutional per case rate of surgical instrument errors via continuous sampling on our healthcare campus. Our third goal was to determine if surgical instrument errors lead to peri-operative/intra-operative delays and to quantify the annual dollars lost in lost chargeable minutes arising from these delays.

## Methods

### Site

This study was conducted at a major, academic, healthcare campus with 36 ORs located across 3 sites (pediatric [7], adult inpatient (23), and adult outpatient [6]). All sites were serviced by the same sterile processing department staff at two facilities in adjacent buildings. **An exemption was granted by the IRB of the University of Wisconsin School of Medicine and Public Health as no patient data was collected and this study was considered a quality improvement study.**

### Observations

Direct observations of surgical instrument errors were conducted over a 7-week period (in the summer of 2021) in the 7 ORs of the children’s hospital which shares sterile processing facilities and staff with the other 29 ORs at the adjacent adult hospital. Observations were conducted only during elective OR days (i.e. Monday through Friday, non-holidays, 7:30 AM (8:30 on Wednesdays) to 5 PM). A total of 24 student observers were trained over a course of 2, two-hour sessions in the identification and recording of the following surgical instrument errors:

#### Missing+

- Missing instrument: missing an instrument that is on the count sheet/not enough of the instruments that are standard for the tray.
- Extra instrument: additional instrument not listed on count sheet; includes instruments in excess of what would be considered standard for the tray.
- Wrong instrument: incorrect instrument in the tray in place of another, usually of similar instrument type; essentially a simultaneous extra instrument and missing instrument.

#### Broken/poorly functioning instrument

- Any damage to an instrument affecting proper use, including sharpness.

#### Tray+

- Packaging failure: misassembled tray, concern for sterility (i.e. lack of filter in rigid tray, correct number of linen wraps, etc.)
- Damaged rigid container: externally damaged rigid container suspicious for integrity, sterility; broken pieces of container internally, concern for sterility.
- Failed sterilization indicator: sterility tab or tape failed to change color after autoclaving.

#### Fleet+

- Fleet: local facility/central core does contain enough of desired resource/instrument/tray or incorrect tray was sent to the OR.
- Fleet turnover/timing: internal sterile processing failed to turnover a low availability resource/instrument/tray in a timely matter.

#### Bioburden+

- Possible bioburden/other debris: unidentified debris in tray, cannot rule out bioburden or compromised integrity of rigid container/linens, concern for sterility.
- Moisture/water in the tray.
- True bioburden: gross blood, tissue, hair, bone, etc.
- Improperly assembled instrument: multipiece instrument either unassembled, inappropriately assembled, or incorrectly assembled in the tray.

#### Transport/handling

- storage or transport of linen wrapped tray results in compromise of sterile container (i.e. removed from shelf and linens ripped open, item found in core improperly stored in a way that would compromise sterility, etc.)

Students were deployed in one of two shifts (7:30 AM (8:30 on Wednesdays) to 12:00 PM or 12:00 PM to 5 PM). Surgical service line, case description, number of errors/case, type of error, presence or absence of a delay and length of delay were recorded for each case. For the purposes of this study, a delay was defined as a pause in the flow of work in the OR. During a case this meant that operative progress of the case stopped until the instrument was available. During set up time prior to the case, this meant that the process of setting up the OR and/or bringing the patient back to the room was paused until the critical instrument or surgical tray was available. Case-type (inpatient versus outpatient) was determined by review of the cases by the surgical faculty.

### Estimation of the charge for an OR minute

The estimated dollar amount of a chargeable OR minute was derived from a mixture of indirect and direct expenses + markup. It was a weighted average at our institution between an upper and lower range ($107.71 to $177.00/minute). This weighting was based on the percentage of inpatient and outpatient cases performed on the campus. Expenses that were included were mid-point salary range labor costs for nurses and surgical technicians, average supply costs for supplies less than $150, equipment use (robot and C-arm etc.) and overhead that includes utilities, and support services (EVS, HR, Legal). This estimate was provided by the institution’s Chief Nursing Officer.

### Annual campus operative case totals

Total number of annual cases (inpatient (IP) and outpatient (OP)) for the campus was provided by the Lead Finance Business Partner for Surgical Services at our institution. Of the three sites, the children’s hospital performed a combination of IP and OP cases in the same OR suite whereas at the adult hospital OP and IP cases were performed at different locations.

### Reporting of errors and delays

To facilitate statistical analysis, results were reported as surgical cases with or without a surgical instrument error (case error rate). Surgical instrument related delays were reported as time lost per case as opposed to time lost per error. Thus, if a case had 5 errors and 3 of these had delays then the time lost per case was the sum of the three delays.

### Statistical analysis

Errors were described by reporting counts and percentages, and were compared between groups using Fisher’s Exact tests when possible, and chi-square tests when the number of rows and/or columns were too large. A multivariable logistic regression model was used to evaluate the impact of multiple factors on the likelihood of errors in a case. All analyses were performed using SAS version 9.4 (SAS Institute, Inc., Cary, NC). *P*-values less than 0.05 were considered as significant.

### Calculations

#### Estimating annual cases with errors

Estimated annual cases with errors for the campus was calculated by dividing the total number of cases with errors for each case-type (IP vs. OP) by the total number of each case-type observed. This generated a percentage of affected cases per case-type (case error rate). The annual number of IP and OP surgical cases for the campus across all three operative locations was then multiplied by the respective (IP or OP) case error rate to achieve an estimate.

#### Estimating annual errors for the campus

Estimated annual errors for the campus was calculated by dividing the total number of errors observed per case-type (IP vs. OP) by the total number of each case-type observed to generate a percentage of errors per case type. This percentage was then multiplied by the annual number of each case-type. Errors/year was the sum of these last two products.

#### Estimating the percent of cases with a delay

An upper and lower range for the percentage of IP and OP cases with a delay was calculated in the following manner: the upper range was determined by dividing the total number of a case-type with a delay by the total number of that case type in which the presence or absence of a delay(s) was explicitly documented. The lower range was determined by dividing the total number of a case-type with delay by the total number of a case-type with an error including cases with errors and no documentation around delays.

#### Calculating the average time lost per case

The average amount of time per case lost to delays was generated by dividing the sum of total time in delays by the number of cases with a delay.

#### Estimating the cost of delays in chargeable minutes

An estimated range of the annual dollar amount of delays in lost chargeable minutes was generated in the following manner: the annual total of each case-type was multiplied by the low and high estimate of the percent of case type with a delay. This was then multiplied by the average amount of time per case lost to delays generating a total annual minutes lost to delays. This was then multiplied by the dollar amount of charges for a single OR minute ($153). The final amount was the sum of the estimated lost charges for OR minutes for both inpatient and outpatient cases.

## Results

Observations were made on 562 total surgical cases. Of these, 558 included the case-type (inpatient 110 (IP) or outpatient 448(OP)). Respectively these represented a sampling of 0.68% of the total annual IP cases (total = 15,950) and 4.06% of the annual total OP cases (total = 11,046) There were 415 cases (73.84%) with no recorded errors and 147 (26.16%) with at least one recorded error (Table 1). Of these 147 cases with errors, 90 had only one error and 57 had two or more errors.

**Table 1:** Surgical instrument errors observed per case

Errors observed/case	IP	OP	NR	total Cases	Percent of cases
0	63	348	4	415	73.84%
1	21	69	0	90	16.01%
2	15	21	0	36	6.41%
3	7	3	0	10	1.78%
4	1	4	0	5	0.89%
5	2	2	0	4	0.71%
6	1	1	0	2	0.36%
**Total**	**110**	**448**	**4**	**562**	**100%**

**Fig. 1:**
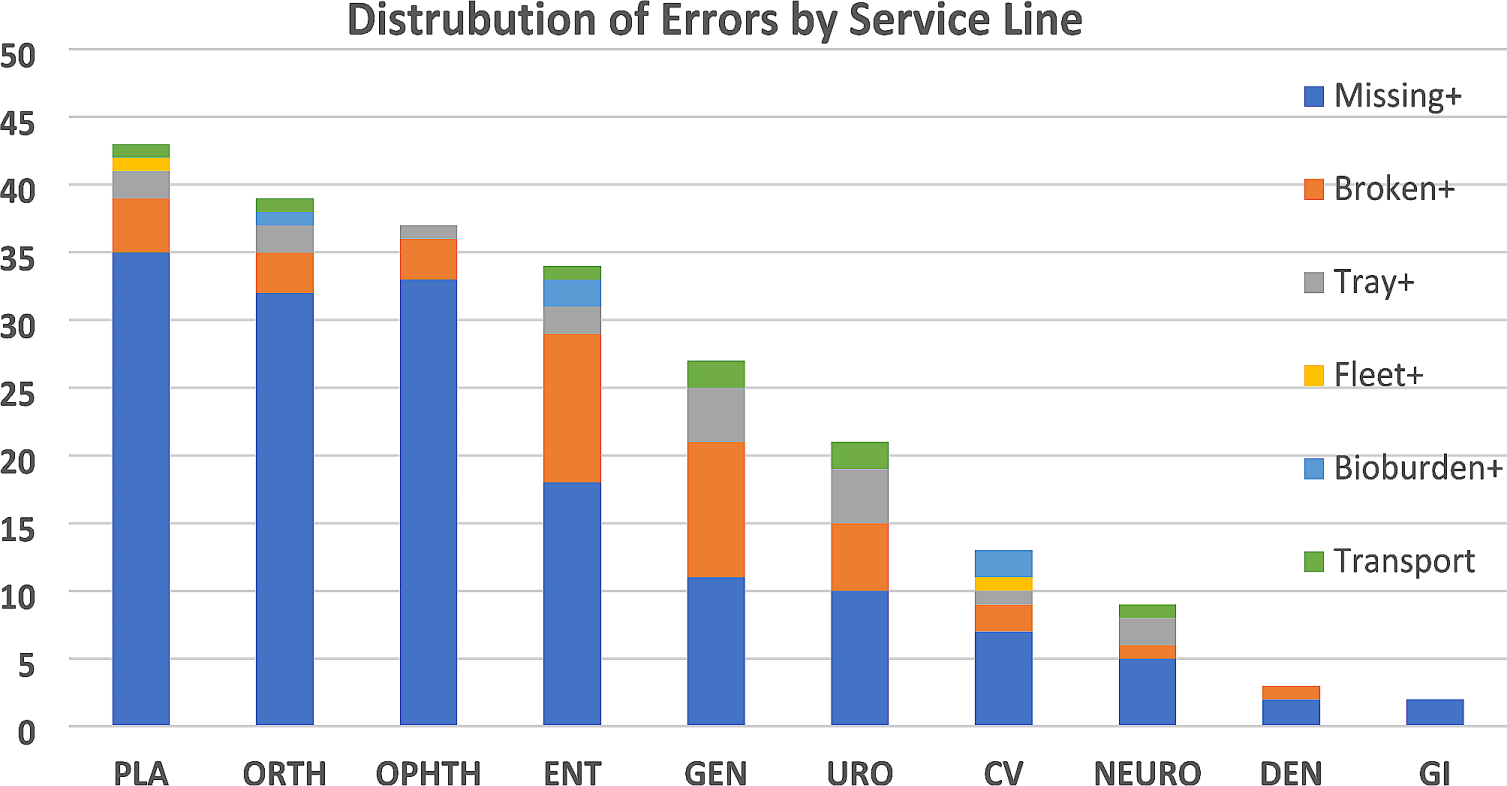
Distribution of Error Types by Service Line. For all service lines, the most common surgical instrument error fell into the Missing + category. PLA, plastics; ORTH, orthopedics; OPHTH, ophthalmology; ENT, otolaryngology; GEN, general; URO, urology; CV, cardiovascular; NEURO, neurosurgery: DEN, dental GI, gastroenterology

The total number of errors over these 147 cases was 236. The most common error-type (Table 2) and the most common error-type across all services lines (Fig. 1) fell into the Missing + category which accounted for 67.8% of all observed errors. Moreover, in accordance with our hypothesis, error types that arise from mistakes in inspection and identification (Missing+/Broken/Bioburden+) accounted for a majority (88.6%) of all observed errors.

**Table 2:** Total observed surgical instrument errors

Error Type	Count of observed errors
**Missing+**	
Missing instrument	144
Wrong instrument	9
Extra instrument	7
***Missing + Total***	**160**
***Broken/poorly functioning instrument Total***	**44**
**Tray+**	
Packaging failure	7
Damaged rigid container	2
Failed sterilization	4
***Tray + Total***	**13**
**Fleet+**	
Fleet	6
Fleet/turnover	1
***Fleet + Total***	**7**
**Bioburden+**	
Possible bioburden/other debris	4
Improperly assembled instrument	1
***Bioburden + Total***	**5**
**Transport/handling**	**7**
***Total***	**236**

We then conducted a multivariable analysis evaluating the impact of IP vs. OP case type (Table 3) and service line (Table 4), using a logistic regression model based on the service lines with at least 20 cases. Both factors (IP vs. OP, *p* = 0.0129 and Service line, *p* = 0.0063) had a significant impact on the chance of observing an error as inpatient cases were significantly more likely to have an error than outpatient cases.

**Table 3:** Surgical cases with errors by case type (IP vs. OP)

Case type	Cases with no errors	Cases with an error	total	percent
Inpatient	63	47	110	42.73%
Outpatient	348	100	448	22.32%
**total**	**411**	**147**	**558**	**26.34%**

**Table 4:** Percent of cases with an error for service lines with a minimum 20 cases

Service Line	Cases with no error	Cases with an error	total cases	% with an error
PLA	27	20	47	42.55%
ORTH	36	24	60	40.00%
OPHTH	52	18	70	25.71%
GEN	54	16	70	22.86%
ENT	99	28	127	22.05%
URO	53	13	66	19.70%
GI	42	2	44	4.55%

PLA, plastics; ORTH, orthopedics; OPHTH, ophthalmology; GEN, general; ENT, otolaryngology; URO, urology; GI, gastroenterology

### Estimating annual cases with errors

To achieve our second goal: to establish an institutional per case rate of surgical instrument errors, we generated an estimate of the total number of surgical cases affected by errors/year occurring across our campus (Table 5). Based on this calculation an estimated 9281 cases (34.37% of all cases) were affected by at least one surgical instrument error.

**Table 5:** Estimated annual cases with errors

Case type	Case error Rate	Annual cases	total cases with errors
Inpatient	0.4273	15,950	6815
Outpatient	0.2232	11,046	2465
Total		26,996	***9281***

### Estimating annual errors for the campus

As 10.15% of all cases observed experienced multiple errors per case, we set out to estimate the total number of errors per year on our campus based on the distribution of errors by case-type and the annual number of IP and OP cases. Based on this calculation there were an estimated 16,311 errors per year throughout our campus (Table 6).

**Table 6:** Estimated annual errors

Case type	Cases observed	Errors observed	Annual cases	Errors/year
Inpatient	110	88	15,950	12,760
Outpatient	448	144	11,046	3551
Total	558	232	26,996	***16,311***

### Calculating the average time lost per case

We then determined the total time lost to delays arising from errors on a per case basis. Only 103 (70.06%) of the 147 cases with errors had specific documentation of whether the error did or did not result in a delay. In the remaining 44 cases there was no documentation. Of the cases for which we had data, inpatient cases were more likely to be associated with a delay (Table 7). There was no statistical difference between the average amount of time per case lost to delays per case type (IP = 11.64 min. vs. OP = 9.98 min., two-tailed, student t-test = 0.780). The average amount of time per case lost to delays for all case-types was 10.16 +/- 21.98 min. This average time lost for all case-types was then used in our subsequent calculations.

**Table 7:** Percent of surgical cases with a delay by case-type (IP vs. OP)

	Variables				Est. range of % delays	
Case-type	Total cases (TC)	Total cases with errors (TCE)	Total cases with delay data (TCDD)	Cases with a delay (CD)	% delays (Low) (CD/TCE) x (TCE/TC)	% delays (High) (CD/TCDD ) x (TCE/TC)
Inpatient	110	47	34	24	21.82%	30.16%
Outpatient	448	100	69	35	7.81%	11.32%

### Estimating the percent of cases with a delay and the cost of delays in chargeable minutes

To achieve our final goal: to quantify the annual cost of these delays in lost chargeable minutes, we generated an estimated range for the annual dollar amount of delays in lost chargeable minutes arising from surgical instrument errors. The first step in this was establishing a range (low-high) for the percent of total observed case-types with a delay (Table 7). We used the low and high estimate of the percent for delays from Table 7 to calculate an annual dollar amount of the lost chargeable minutes due to surgical instrument delays (Table 8). Based on this calculation, the estimated annual dollar amount of lost chargeable minutes for all cases ranged between $6,751,058.06 and $9,421,590.11.

**Table 8:** Annual estimated lost chargeable minutes for entire healthcare campus

Est. % delays (Low)	Est. % delays (High)	Annual Cases	Ave. minutes per delay	Charge per minute	Est. lost charges per year (Low)	Est. lost charges per year (High)
21.82%	30.16%	15,950	10.16	$ 153.00	$ 5,409,590.40	$ 7,477,963.20
7.81%	11.32%	11,046	10.16	$ 153.00	$ 1,341,467.66	$ 1,944,156.03
**Total**					**$ 6,751,058.06**	**$ 9,422,119.23**

## Discussion

This study examines and estimates the rate of, and dollars lost in chargeable minutes from surgical instrument errors in the operating rooms at a major medical facility/campus. Our findings indicate that: (1) Errors arising from failures in visualization/Inspection (Missing+, Broken/poorly functioning, and Bioburden+) account for 88.6% of all observable errors, which is consistent with our hypothesis and previous risk modeling of the surgical instrument cycle [3]. (2) The estimated institutional percentage of surgical cases affected by errors was 34.37%. And (3) many surgical instrument errors result in significant delays and lead to a sizable loss in chargeable OR minutes.

Our previous study, which risk-modeled sterile processing of surgical instruments, indicated that the tasks at highest risk for errors involved visualization [3]. This was based on the complexity and associated risk of error for the four inspection tasks during sterile processing. Consistent with this modeling, our follow-up study on staff reported rates of surgical instrument errors demonstrated that the most commonly reported errors had to do with instrument inspection for functionality, bioburden, and identification for proper sorting into the correct trays [1]. The results of the work presented here are consistent with both the hypothesis laid out in the introduction of this article and our prior modeling. The majority of observed surgical instrument errors in this study (88.6%) fell into the three categories dealing with visualization (Missing+, Broken/poorly functioning, and Bioburden+). The compendium of evidence so far indicates that inspection of surgical instruments is a critically vulnerable task in the sterile processing of surgical instruments that needs to be addressed.

Establishing an institutional rate of surgical instrument errors is critical for several reasons. First, the current staff driven reporting mechanisms appear to greatly underreport these events and do not provide an accurate estimation of percentage of cases in which surgical instrument errors disrupt OR efficiencies and the cost of these disruptions. Our observed rate of cases affected by errors was significantly higher than the staff reported rate at our institution [1]. In that previous study we collected and analyzed a year’s worth of staff reported surgical instrument errors from the Patient Safety Notice (PSN) system. PSNs were filed on 368 of approximately 33,000 cases for a case rate of 1.11%. Second, because observers in this study captured data in real time, we were able to estimate the cost of surgical instrument errors by measuring delays and calculated the number of lost chargeable OR minutes. These two pieces of data (case error rate and time lost) for both inpatient and outpatient cases provide important tools to make granular and accurate estimates of the dollar amount lost in chargeable OR minutes to surgical instrument errors.

Regarding differences in error rates between inpatient and outpatient cases there is the important caveat that this sampling was performed at the children’s hospital so extrapolating this data to the adult hospital, where the majority of cases are inpatient, must be undertaken with caution. It is however generally accepted that inpatient procedures whether in a children’s hospital or an adult hospital are more complex, less iterative, usually longer and require more instruments. All these variables would result in more complex trays with more instruments for inpatient cases and thus a higher case rate of errors. This would hold true even if the error per instrument rate were constant in sterile processing. For example, given a rate of 1 error for every 50 instruments processed, an outpatient procedure that requires one 50 instrument tray versus an inpatient procedure that requires one 100 instrument tray, one would predict a higher percentage of inpatient cases affected. Furthermore, the likelihood of an error in a case would increase with a larger number of surgical trays per case.

There are some significant limitations to this study. First, all data captured was not complete as in 29.9% of cases with errors (8.54% of all cases) had no documentation as to whether there was an associated delay or not. While the rate of incomplete reporting in this study (8.54% of of all cases with incomplete reporting) is less than in our previous study on staff-reported surgical instrument errors (73.7% cases with incomplete reporting) [1], it still represents a limitation of this study. Second, as our primary observers were college students unfamiliar with the OR, we suspect that they failed to capture data on a large percentage of errors that were occurring at the beginning of the study. Thus, we believe the results presented here underestimate the true incidence of surgical instrument errors. Moreover, whether we used inexperienced or experienced observers, humans still make errors at a constant rate and this rate is exacerbated by stress [2]. There is likely a fixed rate of error in making these observations that is inescapable even with the most well trained and experienced humans doing this work. A third limitation is that we estimated the error rates for an entire campus based on only one operative site. For example, the average number of instruments per tray at the adult hospital may be higher than at the children’s hospital. In this case, our predictions would underestimate the real number of errors at the adult hospital [4]. Fourth, we did not capture cases outside of the elective OR window, missing a large proportion of add-on, cases. Thus, we have no way of estimating the dollars lost in chargeable OR minutes in the non-elective/afterhours window. Fifth, the cost of reprocessing additional trays or replacing lost instruments is not included in our calculation simply because we did not collect data on this. Sixth, we were unable to calculate how delays increase the tension in the OR, affect surgical staff performance, well-being, and ultimately the rate of staff attrition. However, there is an abundance of data that environmental stressors erode human performance, increase error rates for all task types, and lead to job burnout [5]. The cost of these impacts is yet to be quantified. Seventh, the accuracy of estimating waste in chargeable minutes is affected by the limitations around accurately estimating the cost of an OR minute. We utilized the cost of an OR minute given to us in good faith by the institution’s leadership. This number of $153/minute is still an estimate. Finally, this study makes an argument for an overhaul of the process of sterile processing on grounds of waste reduction, cost reduction and increased efficiencies. We have no data that directly or indirectly addresses patient outcomes affected by these errors.

While this study is a sampling with the above limitations, there are clear directives that point to specific mitigation strategies. As the majority of observed errors arise from failures in inspection, identification, and sorting of instruments, tasks in sterile processing that involve visualization are at the highest risk for errors. Furthermore, it has been well established in related fields of study that human visual accuracy deteriorates under stress [6–8]. Thus, optimizing the sterile processing space to limit stress is of paramount importance. Application of camera and AI technologies that improve accuracy of inspection and instrument identification need to be implemented as well. Finally, a much as 50–70% of instruments on the average tray go unused during a case [9]. Smart tray technologies and reduction strategies that customize trays to surgeons’ needs would reduce the total number of instruments being processed daily [10]. Such a shift would reduce workloads on staff [4], and likely stress as well as errors and subsequent delays and waste.

## Conclusions

Surgical instrument errors are a significant cause of waste in the form of lost chargeable OR minutes. Our data indicate that most of these errors arise from tasks involving identification, inspection for bioburden and functionality and sorting of instruments into the correct trays. Reduction of these errors will likely require introduction of technologies to limit errors arising from these tasks: specifically smart camera technologies likely paired with AI and/or machine learning.

### Electronic supplementary material

Below is the link to the electronic supplementary material.

Supplementary Material 1

Supplementary Material 2

## Data Availability

Availability of data and materials: The datasets used and/or analyzed during the current study are available from the corresponding author on reasonable request.
