# Supplementary material for: Observed rates of surgical instrument errors point to visualization tasks as being a critically vulnerable point in sterile processing and a significant cause of lost chargeable OR minutes
Source: BMC Surg. 2024 Apr 15;24:110. doi: 10.1186/s12893-024-02407-1 (PMC11017563; doi:10.1186/s12893-024-02407-1)
Supplement: Supplementary file 1 — Supplementary Material 1 [file 12893_2024_2407_MOESM1_ESM.docx]

The SAS System 14:41 Tuesday, February 27, 2024 1

The FREQ Procedure

N_ERRORS ERRORS_YN

Frequency‚N ‚Y ‚ Total

---------ˆ--------ˆ--------ˆ

0 ‚ 415 ‚ 0 ‚ 415

---------ˆ--------ˆ--------ˆ

1 ‚ 0 ‚ 90 ‚ 90

---------ˆ--------ˆ--------ˆ

2 ‚ 0 ‚ 36 ‚ 36

---------ˆ--------ˆ--------ˆ

3 ‚ 0 ‚ 10 ‚ 10

---------ˆ--------ˆ--------ˆ

4 ‚ 0 ‚ 5 ‚ 5

---------ˆ--------ˆ--------ˆ

5 ‚ 0 ‚ 4 ‚ 4

---------ˆ--------ˆ--------ˆ

6 ‚ 0 ‚ 2 ‚ 2

---------ˆ--------ˆ--------ˆ

Total 415 147 562

OP_IP ERRORS_YN

Frequency‚

Row Pct ‚N ‚Y ‚ Total

---------ˆ--------ˆ--------ˆ

IP ‚ 63 ‚ 47 ‚ 110

‚ 57.27 ‚ 42.73 ‚

---------ˆ--------ˆ--------ˆ

OP ‚ 348 ‚ 100 ‚ 448

‚ 77.68 ‚ 22.32 ‚

---------ˆ--------ˆ--------ˆ

Total 411 147 558

Statistics for Table of OP_IP by ERRORS_YN

Statistic DF Value Prob

------------------------------------------------------

Chi-Square 1 18.9520 <.0001

Likelihood Ratio Chi-Square 1 17.6292 <.0001

Continuity Adj. Chi-Square 1 17.9149 <.0001

Mantel-Haenszel Chi-Square 1 18.9180 <.0001

Fisher's Exact Test

----------------------------------

Cell (1,1) Frequency (F) 63

Left-sided Pr <= F <.0001

Right-sided Pr >= F 1.0000

Table Probability (P) <.0001

Two-sided Pr <= P <.0001

The SAS System 14:41 Tuesday, February 27, 2024 3

The FREQ Procedure

SERVICE ERRORS_YN

Frequency ‚

Row Pct ‚N ‚Y ‚ Total

------------ˆ--------ˆ--------ˆ

ENT ‚ 99 ‚ 28 ‚ 127

‚ 77.95 ‚ 22.05 ‚

------------ˆ--------ˆ--------ˆ

GEN ‚ 54 ‚ 16 ‚ 70

‚ 77.14 ‚ 22.86 ‚

------------ˆ--------ˆ--------ˆ

GI ‚ 42 ‚ 2 ‚ 44

‚ 95.45 ‚ 4.55 ‚

------------ˆ--------ˆ--------ˆ

OPHTH ‚ 52 ‚ 18 ‚ 70

‚ 74.29 ‚ 25.71 ‚

------------ˆ--------ˆ--------ˆ

ORTH ‚ 36 ‚ 24 ‚ 60

‚ 60.00 ‚ 40.00 ‚

------------ˆ--------ˆ--------ˆ

PLA ‚ 27 ‚ 20 ‚ 47

‚ 57.45 ‚ 42.55 ‚

------------ˆ--------ˆ--------ˆ

URO ‚ 53 ‚ 13 ‚ 66

‚ 80.30 ‚ 19.70 ‚

------------ˆ--------ˆ--------ˆ

Total 363 121 484

Statistics for Table of SERVICE by ERRORS_YN

Statistic DF Value Prob

------------------------------------------------------

Chi-Square 6 26.5125 0.0002

Likelihood Ratio Chi-Square 6 28.6538 <.0001

Mantel-Haenszel Chi-Square 1 3.5151 0.0608

The SAS System 14:41 Tuesday, February 27, 2024 4

Multivariable model based on services with at least 20 cases

The LOGISTIC Procedure

Model Information

Data Set WORK.LSERVICE

Response Variable ERRORS_YN

Number of Response Levels 2

Model binary logit

Optimization Technique Fisher's scoring

Number of Observations Read 480

Number of Observations Used 480

Response Profile

Ordered Total

Value ERRORS_YN Frequency

1 N 359

2 Y 121

Probability modeled is ERRORS_YN='Y'.

Class Level Information

Class Value Design Variables

OP_IP IP 1

OP 0

SERVICE ENT 1 0 0 0 0 0

GEN 0 1 0 0 0 0

GI 0 0 1 0 0 0

OPHTH 0 0 0 1 0 0

ORTH 0 0 0 0 1 0

PLA 0 0 0 0 0 1

URO 0 0 0 0 0 0

Model Convergence Status

Convergence criterion (GCONV=1E-8) satisfied.

Model Fit Statistics

Intercept

Intercept and

Criterion Only Covariates

AIC 544.028 522.792

SC 548.202 556.182

-2 Log L 542.028 506.792

Testing Global Null Hypothesis: BETA=0

Test Chi-Square DF Pr > ChiSq

Likelihood Ratio 35.2360 7 <.0001

Score 33.8425 7 <.0001

Wald 28.4867 7 0.0002

Type 3 Analysis of Effects

Wald

Effect DF Chi-Square Pr > ChiSq

OP_IP 1 6.1777 0.0129

SERVICE 6 17.9663 0.0063

The SAS System 14:41 Tuesday, February 27, 2024 5

The LOGISTIC Procedure

Analysis of Maximum Likelihood Estimates

Standard Wald

Parameter DF Estimate Error Chi-Square Pr > ChiSq

Intercept 1 -1.4053 0.3095 20.6177 <.0001

OP_IP IP 1 0.8198 0.3298 6.1777 0.0129

SERVICE ENT 1 0.1415 0.3773 0.1406 0.7077

SERVICE GEN 1 -0.2311 0.4629 0.2492 0.6177

SERVICE GI 1 -1.6389 0.7871 4.3359 0.0373

SERVICE OPHTH 1 0.3445 0.4130 0.6956 0.4043

SERVICE ORTH 1 0.6151 0.4404 1.9508 0.1625

SERVICE PLA 1 0.9616 0.4383 4.8142 0.0282

Odds Ratio Estimates

Point 95% Wald

Effect Estimate Confidence Limits

OP_IP IP vs OP 2.270 1.189 4.333

SERVICE ENT vs URO 1.152 0.550 2.413

SERVICE GEN vs URO 0.794 0.320 1.966

SERVICE GI vs URO 0.194 0.042 0.908

SERVICE OPHTH vs URO 1.411 0.628 3.171

SERVICE ORTH vs URO 1.850 0.780 4.386

SERVICE PLA vs URO 2.616 1.108 6.176

Association of Predicted Probabilities and Observed Responses

Percent Concordant 59.8 Somers' D 0.319

Percent Discordant 27.9 Gamma 0.364

Percent Tied 12.4 Tau-a 0.121

Pairs 43439 c 0.659
