# Supplementary material for: Observed rates of surgical instrument errors point to visualization tasks as being a critically vulnerable point in sterile processing and a significant cause of lost chargeable OR minutes
Source: BMC Surg. 2024 Apr 15;24:110. doi: 10.1186/s12893-024-02407-1 (PMC11017563; doi:10.1186/s12893-024-02407-1)
Supplement: Supplementary file 2 — Supplementary Material 2 [file 12893_2024_2407_MOESM2_ESM.docx]

The SAS System 17:21 Monday, February 26, 2024 1

The FREQ Procedure

Cumulative Cumulative

N_ERRORS Frequency Percent Frequency Percent

-------------------------------------------------------------

0 415 73.84 415 73.84

1 90 16.01 505 89.86

2 36 6.41 541 96.26

3 10 1.78 551 98.04

4 5 0.89 556 98.93

5 4 0.71 560 99.64

6 2 0.36 562 100.00

73.8% of cases had no errors.

Looking at this, my suggestion would be to treat errors as present [1-6] or absent [0] in multivariable modeling.

The SAS System 17:21 Monday, February 26, 2024 2

The FREQ Procedure

SERVICE OP_IP

Frequency ‚ ‚IP ‚OP ‚ Total

------------ˆ--------ˆ--------ˆ--------ˆ

‚ 0 ‚ 0 ‚ 2 ‚ 2

------------ˆ--------ˆ--------ˆ--------ˆ

CV ‚ 0 ‚ 13 ‚ 0 ‚ 13

------------ˆ--------ˆ--------ˆ--------ˆ

DEN ‚ 0 ‚ 1 ‚ 17 ‚ 18

------------ˆ--------ˆ--------ˆ--------ˆ

DERM ‚ 0 ‚ 0 ‚ 1 ‚ 1

------------ˆ--------ˆ--------ˆ--------ˆ

ENT ‚ 3 ‚ 4 ‚ 120 ‚ 127

------------ˆ--------ˆ--------ˆ--------ˆ

ENT/DEN ‚ 0 ‚ 0 ‚ 2 ‚ 2

------------ˆ--------ˆ--------ˆ--------ˆ

ENT/DEN/URO ‚ 0 ‚ 0 ‚ 1 ‚ 1

------------ˆ--------ˆ--------ˆ--------ˆ

ENT/GEN ‚ 0 ‚ 0 ‚ 1 ‚ 1

------------ˆ--------ˆ--------ˆ--------ˆ

ENT/GI/PULM ‚ 0 ‚ 0 ‚ 1 ‚ 1

------------ˆ--------ˆ--------ˆ--------ˆ

ENT/OPHTH ‚ 0 ‚ 0 ‚ 2 ‚ 2

------------ˆ--------ˆ--------ˆ--------ˆ

ENT/PULM/GI ‚ 0 ‚ 0 ‚ 2 ‚ 2

------------ˆ--------ˆ--------ˆ--------ˆ

GEN ‚ 0 ‚ 32 ‚ 38 ‚ 70

------------ˆ--------ˆ--------ˆ--------ˆ

GEN/ENT ‚ 0 ‚ 1 ‚ 0 ‚ 1

------------ˆ--------ˆ--------ˆ--------ˆ

GEN/PLA ‚ 0 ‚ 0 ‚ 2 ‚ 2

------------ˆ--------ˆ--------ˆ--------ˆ

GI ‚ 0 ‚ 0 ‚ 44 ‚ 44

------------ˆ--------ˆ--------ˆ--------ˆ

GU ‚ 0 ‚ 0 ‚ 1 ‚ 1

------------ˆ--------ˆ--------ˆ--------ˆ

NEURO ‚ 0 ‚ 16 ‚ 1 ‚ 17

------------ˆ--------ˆ--------ˆ--------ˆ

NEURO/OPHTH ‚ 0 ‚ 1 ‚ 0 ‚ 1

------------ˆ--------ˆ--------ˆ--------ˆ

NEURO/PLA ‚ 0 ‚ 1 ‚ 0 ‚ 1

------------ˆ--------ˆ--------ˆ--------ˆ

OPHTH ‚ 0 ‚ 0 ‚ 70 ‚ 70

------------ˆ--------ˆ--------ˆ--------ˆ

OPHTH/GEN ‚ 0 ‚ 0 ‚ 1 ‚ 1

------------ˆ--------ˆ--------ˆ--------ˆ

OPHTH/PLA ‚ 0 ‚ 0 ‚ 1 ‚ 1

------------ˆ--------ˆ--------ˆ--------ˆ

ORTH ‚ 0 ‚ 27 ‚ 33 ‚ 60

------------ˆ--------ˆ--------ˆ--------ˆ

PEDS GEN ‚ 0 ‚ 1 ‚ 1 ‚ 2

------------ˆ--------ˆ--------ˆ--------ˆ

PLA ‚ 1 ‚ 10 ‚ 36 ‚ 47

------------ˆ--------ˆ--------ˆ--------ˆ

PLA/ENT ‚ 0 ‚ 1 ‚ 1 ‚ 2

------------ˆ--------ˆ--------ˆ--------ˆ

PLA/NEURO ‚ 0 ‚ 2 ‚ 0 ‚ 2

------------ˆ--------ˆ--------ˆ--------ˆ

PULM ‚ 0 ‚ 0 ‚ 2 ‚ 2

------------ˆ--------ˆ--------ˆ--------ˆ

URO ‚ 0 ‚ 0 ‚ 66 ‚ 66

------------ˆ--------ˆ--------ˆ--------ˆ

URO/GI/PLA ‚ 0 ‚ 0 ‚ 1 ‚ 1

------------ˆ--------ˆ--------ˆ--------ˆ

URO/OPHTH ‚ 0 ‚ 0 ‚ 1 ‚ 1

------------ˆ--------ˆ--------ˆ--------ˆ

Total 4 110 448 562

2 cases missing SERVICE [153, 251]

4 cases missing OP_IP [109, 115, 173, 175]

Based on this table, in order to run a multivariable model, we will either need to combine services or run a model on a limited number of services. We could create a “MULTIPLE” services category. That still leaves DERM, GU, PEDS GEN, and PULM with very small sample sizes.

The SAS System 17:21 Monday, February 26, 2024 3

The FREQ Procedure

Table of OP_IP by N_ERRORS

OP_IP N_ERRORS

Frequency‚

Row Pct ‚ 0‚ 1‚ 2‚ 3‚ 4‚ 5‚ 6‚ Total

---------ˆ--------ˆ--------ˆ--------ˆ--------ˆ--------ˆ--------ˆ--------ˆ

‚ 4 ‚ 0 ‚ 0 ‚ 0 ‚ 0 ‚ 0 ‚ 0 ‚ 4

‚ 100.00 ‚ 0.00 ‚ 0.00 ‚ 0.00 ‚ 0.00 ‚ 0.00 ‚ 0.00 ‚

---------ˆ--------ˆ--------ˆ--------ˆ--------ˆ--------ˆ--------ˆ--------ˆ

IP ‚ 63 ‚ 21 ‚ 15 ‚ 7 ‚ 1 ‚ 2 ‚ 1 ‚ 110

‚ 57.27 ‚ 19.09 ‚ 13.64 ‚ 6.36 ‚ 0.91 ‚ 1.82 ‚ 0.91 ‚

---------ˆ--------ˆ--------ˆ--------ˆ--------ˆ--------ˆ--------ˆ--------ˆ

OP ‚ 348 ‚ 69 ‚ 21 ‚ 3 ‚ 4 ‚ 2 ‚ 1 ‚ 448

‚ 77.68 ‚ 15.40 ‚ 4.69 ‚ 0.67 ‚ 0.89 ‚ 0.45 ‚ 0.22 ‚

---------ˆ--------ˆ--------ˆ--------ˆ--------ˆ--------ˆ--------ˆ--------ˆ

Total 415 90 36 10 5 4 2 562

As you stated, there is definitely a trend here, with more errors in the IP cases.

The SAS System 17:21 Monday, February 26, 2024 4

The FREQ Procedure

SERVICE N_ERRORS

Frequency ‚

Row Pct ‚ 0‚ 1‚ 2‚ 3‚ 4‚ 5‚ 6‚ Total

------------ˆ--------ˆ--------ˆ--------ˆ--------ˆ--------ˆ--------ˆ--------ˆ

‚ 0 ‚ 2 ‚ 0 ‚ 0 ‚ 0 ‚ 0 ‚ 0 ‚ 2

‚ 0.00 ‚ 100.00 ‚ 0.00 ‚ 0.00 ‚ 0.00 ‚ 0.00 ‚ 0.00 ‚

------------ˆ--------ˆ--------ˆ--------ˆ--------ˆ--------ˆ--------ˆ--------ˆ

CV ‚ 6 ‚ 4 ‚ 1 ‚ 2 ‚ 0 ‚ 0 ‚ 0 ‚ 13

‚ 46.15 ‚ 30.77 ‚ 7.69 ‚ 15.38 ‚ 0.00 ‚ 0.00 ‚ 0.00 ‚

------------ˆ--------ˆ--------ˆ--------ˆ--------ˆ--------ˆ--------ˆ--------ˆ

DEN ‚ 15 ‚ 3 ‚ 0 ‚ 0 ‚ 0 ‚ 0 ‚ 0 ‚ 18

‚ 83.33 ‚ 16.67 ‚ 0.00 ‚ 0.00 ‚ 0.00 ‚ 0.00 ‚ 0.00 ‚

------------ˆ--------ˆ--------ˆ--------ˆ--------ˆ--------ˆ--------ˆ--------ˆ

DERM ‚ 1 ‚ 0 ‚ 0 ‚ 0 ‚ 0 ‚ 0 ‚ 0 ‚ 1

‚ 100.00 ‚ 0.00 ‚ 0.00 ‚ 0.00 ‚ 0.00 ‚ 0.00 ‚ 0.00 ‚

------------ˆ--------ˆ--------ˆ--------ˆ--------ˆ--------ˆ--------ˆ--------ˆ

ENT ‚ 99 ‚ 20 ‚ 7 ‚ 0 ‚ 1 ‚ 0 ‚ 0 ‚ 127 [120/127 94.5% OP]

‚ 77.95 ‚ 15.75 ‚ 5.51 ‚ 0.00 ‚ 0.79 ‚ 0.00 ‚ 0.00 ‚

------------ˆ--------ˆ--------ˆ--------ˆ--------ˆ--------ˆ--------ˆ--------ˆ

ENT/DEN ‚ 2 ‚ 0 ‚ 0 ‚ 0 ‚ 0 ‚ 0 ‚ 0 ‚ 2

‚ 100.00 ‚ 0.00 ‚ 0.00 ‚ 0.00 ‚ 0.00 ‚ 0.00 ‚ 0.00 ‚

------------ˆ--------ˆ--------ˆ--------ˆ--------ˆ--------ˆ--------ˆ--------ˆ

ENT/DEN/URO ‚ 1 ‚ 0 ‚ 0 ‚ 0 ‚ 0 ‚ 0 ‚ 0 ‚ 1

‚ 100.00 ‚ 0.00 ‚ 0.00 ‚ 0.00 ‚ 0.00 ‚ 0.00 ‚ 0.00 ‚

------------ˆ--------ˆ--------ˆ--------ˆ--------ˆ--------ˆ--------ˆ--------ˆ

ENT/GEN ‚ 1 ‚ 0 ‚ 0 ‚ 0 ‚ 0 ‚ 0 ‚ 0 ‚ 1

‚ 100.00 ‚ 0.00 ‚ 0.00 ‚ 0.00 ‚ 0.00 ‚ 0.00 ‚ 0.00 ‚

------------ˆ--------ˆ--------ˆ--------ˆ--------ˆ--------ˆ--------ˆ--------ˆ

ENT/GI/PULM ‚ 1 ‚ 0 ‚ 0 ‚ 0 ‚ 0 ‚ 0 ‚ 0 ‚ 1

‚ 100.00 ‚ 0.00 ‚ 0.00 ‚ 0.00 ‚ 0.00 ‚ 0.00 ‚ 0.00 ‚

------------ˆ--------ˆ--------ˆ--------ˆ--------ˆ--------ˆ--------ˆ--------ˆ

ENT/OPHTH ‚ 2 ‚ 0 ‚ 0 ‚ 0 ‚ 0 ‚ 0 ‚ 0 ‚ 2

‚ 100.00 ‚ 0.00 ‚ 0.00 ‚ 0.00 ‚ 0.00 ‚ 0.00 ‚ 0.00 ‚

------------ˆ--------ˆ--------ˆ--------ˆ--------ˆ--------ˆ--------ˆ--------ˆ

ENT/PULM/GI ‚ 2 ‚ 0 ‚ 0 ‚ 0 ‚ 0 ‚ 0 ‚ 0 ‚ 2

‚ 100.00 ‚ 0.00 ‚ 0.00 ‚ 0.00 ‚ 0.00 ‚ 0.00 ‚ 0.00 ‚

------------ˆ--------ˆ--------ˆ--------ˆ--------ˆ--------ˆ--------ˆ--------ˆ

GEN ‚ 54 ‚ 9 ‚ 4 ‚ 3 ‚ 0 ‚ 0 ‚ 0 ‚ 70 [38/70 54.3% OP]

‚ 77.14 ‚ 12.86 ‚ 5.71 ‚ 4.29 ‚ 0.00 ‚ 0.00 ‚ 0.00 ‚

------------ˆ--------ˆ--------ˆ--------ˆ--------ˆ--------ˆ--------ˆ--------ˆ

GEN/ENT ‚ 1 ‚ 0 ‚ 0 ‚ 0 ‚ 0 ‚ 0 ‚ 0 ‚ 1

‚ 100.00 ‚ 0.00 ‚ 0.00 ‚ 0.00 ‚ 0.00 ‚ 0.00 ‚ 0.00 ‚

------------ˆ--------ˆ--------ˆ--------ˆ--------ˆ--------ˆ--------ˆ--------ˆ

GEN/PLA ‚ 2 ‚ 0 ‚ 0 ‚ 0 ‚ 0 ‚ 0 ‚ 0 ‚ 2

‚ 100.00 ‚ 0.00 ‚ 0.00 ‚ 0.00 ‚ 0.00 ‚ 0.00 ‚ 0.00 ‚

------------ˆ--------ˆ--------ˆ--------ˆ--------ˆ--------ˆ--------ˆ--------ˆ

GI ‚ 42 ‚ 2 ‚ 0 ‚ 0 ‚ 0 ‚ 0 ‚ 0 ‚ 44 [44/44 100% OP]

‚ 95.45 ‚ 4.55 ‚ 0.00 ‚ 0.00 ‚ 0.00 ‚ 0.00 ‚ 0.00 ‚

------------ˆ--------ˆ--------ˆ--------ˆ--------ˆ--------ˆ--------ˆ--------ˆ

GU ‚ 0 ‚ 0 ‚ 1 ‚ 0 ‚ 0 ‚ 0 ‚ 0 ‚ 1

‚ 0.00 ‚ 0.00 ‚ 100.00 ‚ 0.00 ‚ 0.00 ‚ 0.00 ‚ 0.00 ‚

------------ˆ--------ˆ--------ˆ--------ˆ--------ˆ--------ˆ--------ˆ--------ˆ

NEURO ‚ 11 ‚ 5 ‚ 0 ‚ 0 ‚ 1 ‚ 0 ‚ 0 ‚ 17

‚ 64.71 ‚ 29.41 ‚ 0.00 ‚ 0.00 ‚ 5.88 ‚ 0.00 ‚ 0.00 ‚

------------ˆ--------ˆ--------ˆ--------ˆ--------ˆ--------ˆ--------ˆ--------ˆ

NEURO/OPHTH ‚ 1 ‚ 0 ‚ 0 ‚ 0 ‚ 0 ‚ 0 ‚ 0 ‚ 1

‚ 100.00 ‚ 0.00 ‚ 0.00 ‚ 0.00 ‚ 0.00 ‚ 0.00 ‚ 0.00 ‚

------------ˆ--------ˆ--------ˆ--------ˆ--------ˆ--------ˆ--------ˆ--------ˆ

NEURO/PLA ‚ 0 ‚ 0 ‚ 1 ‚ 0 ‚ 0 ‚ 0 ‚ 0 ‚ 1

‚ 0.00 ‚ 0.00 ‚ 100.00 ‚ 0.00 ‚ 0.00 ‚ 0.00 ‚ 0.00 ‚

------------ˆ--------ˆ--------ˆ--------ˆ--------ˆ--------ˆ--------ˆ--------ˆ

OPHTH ‚ 52 ‚ 10 ‚ 3 ‚ 2 ‚ 2 ‚ 0 ‚ 1 ‚ 70 [70/70 100% OP]

‚ 74.29 ‚ 14.29 ‚ 4.29 ‚ 2.86 ‚ 2.86 ‚ 0.00 ‚ 1.43 ‚

------------ˆ--------ˆ--------ˆ--------ˆ--------ˆ--------ˆ--------ˆ--------ˆ

OPHTH/GEN ‚ 0 ‚ 1 ‚ 0 ‚ 0 ‚ 0 ‚ 0 ‚ 0 ‚ 1

‚ 0.00 ‚ 100.00 ‚ 0.00 ‚ 0.00 ‚ 0.00 ‚ 0.00 ‚ 0.00 ‚

------------ˆ--------ˆ--------ˆ--------ˆ--------ˆ--------ˆ--------ˆ--------ˆ

OPHTH/PLA ‚ 0 ‚ 0 ‚ 1 ‚ 0 ‚ 0 ‚ 0 ‚ 0 ‚ 1

‚ 0.00 ‚ 0.00 ‚ 100.00 ‚ 0.00 ‚ 0.00 ‚ 0.00 ‚ 0.00 ‚

------------ˆ--------ˆ--------ˆ--------ˆ--------ˆ--------ˆ--------ˆ--------ˆ

Total 415 90 36 10 5 4 2 562

(Continued)

The SAS System 17:21 Monday, February 26, 2024 5

The FREQ Procedure

SERVICE N_ERRORS

Frequency ‚

Row Pct ‚ 0‚ 1‚ 2‚ 3‚ 4‚ 5‚ 6‚ Total

------------ˆ--------ˆ--------ˆ--------ˆ--------ˆ--------ˆ--------ˆ--------ˆ

ORTH ‚ 36 ‚ 15 ‚ 5 ‚ 2 ‚ 1 ‚ 1 ‚ 0 ‚ 60 [33/60 55% OP]

‚ 60.00 ‚ 25.00 ‚ 8.33 ‚ 3.33 ‚ 1.67 ‚ 1.67 ‚ 0.00 ‚

------------ˆ--------ˆ--------ˆ--------ˆ--------ˆ--------ˆ--------ˆ--------ˆ

PEDS GEN ‚ 2 ‚ 0 ‚ 0 ‚ 0 ‚ 0 ‚ 0 ‚ 0 ‚ 2

‚ 100.00 ‚ 0.00 ‚ 0.00 ‚ 0.00 ‚ 0.00 ‚ 0.00 ‚ 0.00 ‚

------------ˆ--------ˆ--------ˆ--------ˆ--------ˆ--------ˆ--------ˆ--------ˆ

PLA ‚ 27 ‚ 9 ‚ 7 ‚ 0 ‚ 0 ‚ 3 ‚ 1 ‚ 47 [36/47 76.6% OP]

‚ 57.45 ‚ 19.15 ‚ 14.89 ‚ 0.00 ‚ 0.00 ‚ 6.38 ‚ 2.13 ‚

------------ˆ--------ˆ--------ˆ--------ˆ--------ˆ--------ˆ--------ˆ--------ˆ

PLA/ENT ‚ 1 ‚ 0 ‚ 1 ‚ 0 ‚ 0 ‚ 0 ‚ 0 ‚ 2

‚ 50.00 ‚ 0.00 ‚ 50.00 ‚ 0.00 ‚ 0.00 ‚ 0.00 ‚ 0.00 ‚

------------ˆ--------ˆ--------ˆ--------ˆ--------ˆ--------ˆ--------ˆ--------ˆ

PLA/NEURO ‚ 0 ‚ 1 ‚ 1 ‚ 0 ‚ 0 ‚ 0 ‚ 0 ‚ 2

‚ 0.00 ‚ 50.00 ‚ 50.00 ‚ 0.00 ‚ 0.00 ‚ 0.00 ‚ 0.00 ‚

------------ˆ--------ˆ--------ˆ--------ˆ--------ˆ--------ˆ--------ˆ--------ˆ

PULM ‚ 2 ‚ 0 ‚ 0 ‚ 0 ‚ 0 ‚ 0 ‚ 0 ‚ 2

‚ 100.00 ‚ 0.00 ‚ 0.00 ‚ 0.00 ‚ 0.00 ‚ 0.00 ‚ 0.00 ‚

------------ˆ--------ˆ--------ˆ--------ˆ--------ˆ--------ˆ--------ˆ--------ˆ

URO ‚ 53 ‚ 8 ‚ 4 ‚ 1 ‚ 0 ‚ 0 ‚ 0 ‚ 66 [66/66 100% OP]

‚ 80.30 ‚ 12.12 ‚ 6.06 ‚ 1.52 ‚ 0.00 ‚ 0.00 ‚ 0.00 ‚

------------ˆ--------ˆ--------ˆ--------ˆ--------ˆ--------ˆ--------ˆ--------ˆ

URO/GI/PLA ‚ 0 ‚ 1 ‚ 0 ‚ 0 ‚ 0 ‚ 0 ‚ 0 ‚ 1

‚ 0.00 ‚ 100.00 ‚ 0.00 ‚ 0.00 ‚ 0.00 ‚ 0.00 ‚ 0.00 ‚

------------ˆ--------ˆ--------ˆ--------ˆ--------ˆ--------ˆ--------ˆ--------ˆ

URO/OPHTH ‚ 1 ‚ 0 ‚ 0 ‚ 0 ‚ 0 ‚ 0 ‚ 0 ‚ 1

‚ 100.00 ‚ 0.00 ‚ 0.00 ‚ 0.00 ‚ 0.00 ‚ 0.00 ‚ 0.00 ‚

------------ˆ--------ˆ--------ˆ--------ˆ--------ˆ--------ˆ--------ˆ--------ˆ

Total 415 90 36 10 5 4 2 562

I’m not sure where to focus my attention here, since so many sample sizes are small. Of the large case number services (highlighted), the two services with the highest percentage of cases with errors [ORTHO, 40% of cases had errors] and [PLASTICS, 43% of cases had errors] also had a relatively high percentage of IP cases [ORTHO, 45% IP] and {PLASTICS, 23% IP]. However, the service with the highest percentage of IP cases was GENERAL surgery [46% IP], and their percentage of cases with errors [23%] was similar to several of the services with a low percentage of IP cases [ENT, 22% with errors, 5% IP] [ophthalmology, 26% with errors, 0% IP] [urology, 20% with errors, 0% IP].

Here are the large case number services sorted by percentage of cases with errors.

ERR IP

PLA 43% 23%

ORTH 40% 45%

OPHTH 26% 0%

GEN 23% 46%

ENT 22% 5%

URO 20% 0%

GI 5% 0%

Let me know if you would like me to create a multivariable model based on these 7 services, create an 8^th^ “multiple” service, or take some other approach.
